# Environmental context shapes the relationship between grass consumption and body size in African herbivore communities

**DOI:** 10.1002/ece3.11050

**Published:** 2024-02-15

**Authors:** Joel O. Abraham, John Rowan, Kaedan O'Brien, Kathryn G. Sokolowski, J. Tyler Faith

**Affiliations:** ^1^ Department of Ecology and Evolutionary Biology Princeton University Princeton New Jersey USA; ^2^ Department of Anthropology University at Albany Albany New York USA; ^3^ Department of Anthropology University of Utah Salt Lake City Utah USA; ^4^ Natural History Museum of Utah University of Utah Salt Lake City Utah USA; ^5^ Origins Centre University of the Witwatersrand Johannesburg South Africa

**Keywords:** African savannas, body mass, community assembly, diet, grass dependence, Jarman‐Bell principle, large mammalian herbivores, metabolic theory, optimal foraging theory

## Abstract

Though herbivore grass dependence has been shown to increase with body size across herbivore species, it is unclear whether this relationship holds at the community level. Here we evaluate whether grass consumption scales positively with body size within African large mammalian herbivore communities and how this relationship varies with environmental context. We used stable carbon isotope and community occurrence data to investigate how grass dependence scales with body size within 23 savanna herbivore communities throughout eastern and central Africa. We found that dietary grass fraction increased with body size for the majority of herbivore communities considered, especially when complete community data were available. However, the slope of this relationship varied, and rainfall seasonality and elephant presence were key drivers of the variation—grass dependence increased less strongly with body size where rainfall was more seasonal and where elephants were present. We found also that the dependence of the herbivore community as a whole on grass peaked at intermediate woody cover. Intraspecific diet variation contributed to these community‐level patterns: common hippopotamus (*Hippopotamus amphibius*) and giraffe (*Giraffa camelopardalis*) ate less grass where rainfall was more seasonal, whereas Cape buffalo (*Syncerus caffer*) and savanna elephant (*Loxodonta africana*) grass consumption were parabolically related to woody cover. Our results indicate that general rules appear to govern herbivore community assembly, though some aspects of herbivore foraging behavior depend upon local environmental context.

## Graphical abstract

Though herbivore grass dependence has been shown to increase with body size across herbivore species, it is unclear whether this relationship varies between communities in relation to environmental variation. Here we use stable carbon isotope and community occurrence data to investigate how grass dependence scales with body size within herbivore communities throughout eastern and central Africa. We found that dietary grass fraction increased with body size for the majority of herbivore communities considered, but the magnitude of this relationship varied considerably with environmental context, underscoring the fact that, though general rules appear to govern herbivore foraging ecology, some aspects of foraging behavior are indeed dynamic.

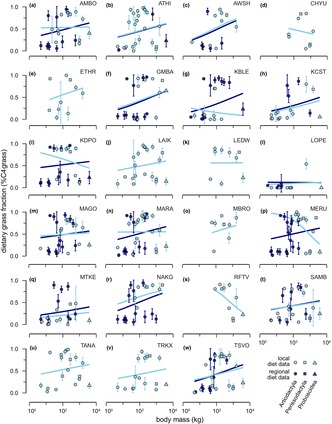

## 1 INTRODUCTION

Do general rules govern community assembly? Within some of the hyperdiverse large mammalian herbivore communities that occupy African savannas, it has been observed that larger species tend to consume more grass (Bell, 1971; Cerling et al., 2003; Jarman, 1974), a pattern that has also been shown to hold across mammalian herbivore species in the tropics (Abraham et al., 2022; Gagnon & Chew, 2000). Two potential mechanisms have been suggested to explain this relationship. The first derives from optimal foraging theory and hinges on differences in the distributions of plant functional types (e.g., trees and grasses) and how plant distributions scale with body size (Bhat et al., 2020; Hempson, Illius, et al., 2015; Staver, 2018). At small body sizes, all food appears patchily distributed, so search costs and the risk of starvation are always high. At large body sizes, in contrast, the distribution of browse remains highly clustered (Staver, 2018; Staver, Asner, et al., 2019), whereas grass is distributed more homogeneously (at least in grass‐dominated systems) (Bhat et al., 2020; Staver, 2018). As such, large herbivores can minimize search costs and therefore lessen starvation risk by specializing in grass (Bhat et al., 2020). The second mechanism derives from metabolic theory, which suggests that small herbivores require high‐quality forage but in small quantities, whereas large herbivores can tolerate lower‐quality forage but require large quantities of it (the Jarman‐Bell Principle; Bell, 1971; Hopcraft et al., 2010; Jarman, 1974; Olff et al., 2002; Potter & Pringle, 2023). Because woody plants have steeper nutrient gradients across tissues than do grasses (Güsewell, 2004; Reich & Oleksyn, 2004), small herbivores can meet their nutritional needs by selectively feeding on only the high‐quality parts of woody plants, such as fruit, buds, and young leaves. In contrast, larger herbivores can satisfy their greater forage requirements by eating grass in bulk (Abraham et al., 2022; Potter & Pringle, 2023). Regardless of the mechanism, the pattern of increasing grass dependence with increasing body size has emerged as a robust pattern across tropical herbivore species (Abraham et al., 2022).

Though this pattern was initially noted at the community level (Bell, 1971; Cerling et al., 2003; Jarman, 1974), it is unclear whether a positive relationship between body size and grass consumption is ubiquitous across savanna herbivore communities. It is well‐documented that the composition of herbivores' diets vary considerably within species across space (Cerling et al., 2003, 2015; Codron et al., 2007; Pansu et al., 2022; Robinson et al., 2021), as does herbivore community composition: not all species are present in every community (Hempson, Archibald, & Bond, 2015; Lomolino et al., 2010; Rowan et al., 2020). As a result, though body mass and dietary grass fraction are correlated across herbivore species in the tropics (Abraham et al., 2022) and within certain communities (Bell, 1971; Cerling et al., 2003; Jarman, 1974), these characteristics might be decoupled within communities that differ in local environmental context.

Indeed, it is probable that the strength (and possibly even the direction) of the relationship should vary between herbivore communities. Both hypothesized mechanisms for this relationship implicate plant characteristics—either their spatial distributions (per optimal foraging theory) or relative quality (per metabolic theory)—in driving the relationship, characteristics that vary substantially across the tropics (Olff et al., 2002; Reich & Oleksyn, 2004; Staver, 2018; Staver et al., 2011). Across the tropics, the availability and quality of plant functional types are highly dependent on environmental factors, such as rainfall, temperature, and the seasonality of both (Güsewell, 2004; Hempson, Archibald, et al., 2015; Olff et al., 2002; Reich & Oleksyn, 2004; Sala et al., 2012; Scanlon et al., 2005). As such, the strength, and possibly direction, of the relationship between body size and grass consumption should likewise vary across herbivore communities in relation to environmental context, further bolstering the predictability of community assembly.

**FIGURE 1:**
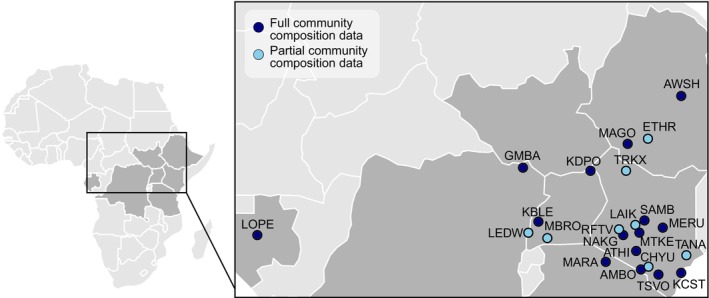
Locations of eastern and central African herbivore communities (*N* = 23) included in this study: AMBO, Amboseli; ATHI, Athi Plains and Nairobi; AWSH, Awash; CHYU, Chyulu Hills; ETHR, Ethiopian Rift Lakes; GMBA, Garamba; KBLE, Kibale; KCST, Kenya Coast; KDPO, Kidepo; LAIK, Laikipia; LEDW, Lake Edward; LOPE, Lopé; MAGO, Omo/Mago; MARA, Maasai Mara‐Serengeti; MBRO, Lake Mburo; MERU, Meru National Park; MTKE, Mount Kenya; NAKG, Nakuru; RFTV, Kenya Rift Valley; SAMB, Samburu; TANA, Tana River; TRKX, Turkana; TSVO, Tsavo. Herbivore communities span seven countries within central and eastern Africa. Local diet data for these communities were available from Cerling et al. (2015) but data were lacking for some species in particular communities. Using community occurrence data from Rowan et al. (2020), we used regional diet estimates to fill in diet data for missing taxa, though this was only possible for the 15 communities which overlapped between the two datasets, represented here by *dark blue points*. Communities represented by *light blue points* were absent from our community composition dataset, and therefore herbivore community data may be incomplete.

To determine the relationship between body size and grass consumption at the community level, we combined local diet data derived from stable carbon isotope data (from Cerling et al., 2015) with species occurrence data (from Rowan et al., 2020) for herbivore communities throughout eastern and central Africa (Figure 1). We then evaluated the factors that best predict variation in the relationship between body size and grass dependence across these communities, revealing insights into herbivore foraging ecology and community assembly.

## 2 MATERIALS AND METHODS

### 2.1 Local diet data

We obtained local diet data from a published stable isotope dataset for large mammalian herbivores (orders Artiodactyla, Perissodactyla, and Proboscidea) from across central and eastern Africa (Cerling et al., 2015). This dataset contains isotope data from 1917 specimens collected between 1896 and 2012 from 59 species across 30 communities. These communities were predefined in the dataset and correspond to single or multiple adjacent protected areas, including national parks and preserves. For four of these communities, Athi Plains (ATHI) and Nairobi National Park (NBNP) as well as Turkana grassland (TRKG) and Turkana regional (TRKX), sample localities were contiguous and/or nested, so these communities were merged: Nairobi National Park was merged with Athi Plains and Turkana grassland was merged into Turkana regional, reducing the number of communities to 28.

From these stable isotope data, we were able to calculate local grass dependence for herbivore species in each community. Stable isotope data can be used to reconstruct animal feeding habits so long as dietary functional groups of interest are sufficiently differentiated isotopically (Cerling et al., 2015; Codron et al., 2007; Rowan et al., 2017). In tropical Africa, nearly all low‐elevation (<2000 m) grasses employ the C_4_ photosynthetic pathway (Edwards et al., 2010), whereas other plant functional groups—trees, shrubs, and forbs—predominantly employ the C_3_ photosynthetic pathway (Cerling et al., 2015; Codron et al., 2007; Rowan et al., 2017). These two photosynthetic pathways fractionate CO_2_ differently during the process of carbon fixation, such that C_3_ plants have a strongly negative carbon isotopic signature (δ^13^C) relative to atmospheric CO_2_, about −26.6‰ for tropical African C_3_ plants, whereas the δ^13^C for C_4_‐photosynthesizing plants, while still negative, is less extreme, at about −10.0‰ for tropical African C_4_ grasses (Ambrose & DeNiro, 1986; Cerling et al., 2015; Cerling & Harris, 1999; Codron et al., 2007; Rowan et al., 2017). The isotopic differences between plant functional groups are preserved in the isotopic composition of the herbivores that consume them (Ambrose & DeNiro, 1986; Cerling et al., 2015; Cerling & Harris, 1999; Rowan et al., 2017). Thus, throughout much of tropical Africa where plant functional groups employ distinct photosynthetic pathways, it is possible to reconstruct the relative proportions of C_4_ versus C_3_ plants (and therefore grasses vs. non‐grasses) consumed by herbivores via stable carbon isotope analysis of their tissues.

Following Cerling and others (Cerling et al., 2015; Robinson et al., 2021), we analyzed the δ^13^C_enamel_‐equivalent values in the dataset, which included converted δ^13^C_hair‐keratin_ and δ^13^C_collagen_ samples. The relative contributions of C_3_ and C_4_ plants to diet were then estimated from the δ^13^C_enamel_ values using a dual endpoint mixing model with known C_3_ and C_4_ plant end member δ^13^C values (Abraham et al., 2019; Cerling et al., 2015; Codron et al., 2007; Rowan et al., 2017):

$$\delta^{13}C_{\text{measured}}-ɛ=\delta^{13}{C_{C}}_{3}\times {f_{C}}_{3}+\delta^{13}{C_{C}}_{4}\times {f_{C}}_{4},$$

where ${f_{C}}_{3}$ and ${f_{C}}_{4}$ correspond to the fractions of C_3_ and C_4_ plants consumed respectively. We assumed an enrichment factor *ɛ* of +14.1‰ between diet and tooth enamel, per previous studies (see Cerling & Harris, 1999). The C_3_ and C_4_ endmembers we employed here were calculated based on mean values for plants and were −26.6‰ and −10.0‰ for C_3_ and C_4_ plant biomass respectively, corresponding to δ^13^C_enamel_ values of −12.5‰ and 4.1‰ (Cerling et al., 2015; Rowan et al., 2017).

Once isotope values had been converted to dietary grass fraction estimates, we then calculated local estimates of dietary grass fraction for every species within each community as well as corresponding standard deviations (see Appendix S1). To account for within‐site variation in species' diets and to capture uncertainty in diet estimates, mean dietary grass fraction estimates were weighted by their corresponding standard deviations in all analyses (see Section 2.4 below).

Because most grasses in Africa are C_4_‐photosynthesizing and other plants employing the C_4_ photosynthetic pathway are relatively rare (Edwards et al., 2010), the above approach reliably recapitulates dietary grass fraction for herbivores throughout most of tropical Africa (Cerling et al., 2015; Codron et al., 2007; Robinson et al., 2021). However, within some high‐altitude (>2000 m) regions in tropical Africa, C_3_‐photosynthesizing grasses are present (Edwards et al., 2010), making it impossible to isolate the grass component of herbivore diets from stable isotope analysis alone. Three of the communities in the isotope dataset—Aberdare (ABER), Bale Mountains (BALE), and Simien Mountains (SIME)—are at sufficiently high elevations such that C_3_ grasses are prevalent and dietary grass fraction therefore cannot be reliably estimated from stable isotope data. Thus, to avoid introducing directional bias into our analyses by underestimating species' grass consumption in these communities, these three high‐elevation communities were excluded from our analyses.

In addition, for two communities, Ituri forest (ITRI) and Kahuzi‐Biega (KZBG), stable isotope data indicated that all herbivore species therein were pure browsers (dietary grass fraction = 0). Indeed, Ituri forest and Kahuzi‐Biega are both closed‐canopy forest sites wherein grass is scarce. Consequently, these two communities were also removed from our analyses, as there was no within‐community diet variation, and calculating a slope between dietary grass fraction and body mass would therefore have been inappropriate. No other communities had any obvious reason to be excluded from analyses and were therefore retained.

With these five communities excluded, the filtered isotope dataset included 1493 samples representing 46 species across 23 communities (Figure 1).

### 2.2 Herbivore community composition data

Though extensive, this stable isotope dataset lacked local data for some species known to occur within the 23 communities. To determine which species were present in a particular community but lacking isotope data, we used a dataset of herbivore community composition across Africa (Rowan et al., 2020). These herbivore community composition data were derived from field surveys, species lists, and local databases (Rowan et al., 2020). Community data were cross‐checked with multiple sources, including species range maps derived from the International Union for Conservation of Nature (IUCN), and were standardized to the IUCN's Red List taxonomy (Rowan et al., 2020). In some cases, however, local isotope data for a species within a particular community were available in the stable isotope dataset but that species was not listed as present in the community composition dataset. In such instances, that species was considered to be present in the community and its isotope data were retained.

Fifteen of the 23 communities included in our analyses were present in the community composition dataset; for the other eight communities included in the isotopic dataset but not present in the community composition dataset, community data could not be assumed to be complete (Figure 1). Within the 15 communities overlapping between the two datasets, local isotope data were lacking for 155 community members. To derive diet grass fraction estimates for these community members, we calculated regional species‐level dietary grass fraction averages—the mean dietary grass fraction of all samples for a given species across all 23 sites—as well as corresponding standard deviations from the stable isotope dataset. We used those regional diet estimates as the dietary grass fraction values for those taxa missing local diet data within the 15 communities shared between the two datasets only; community‐specific diet estimates derived from local isotope data were retained for all other community members (see Appendix S1).

Some species present in the communities (*N* = 11; see Appendix S1) were missing from the stable isotope dataset altogether, such that it was not possible to calculate regional averages for those species from the stable isotope dataset. Global estimates of dietary grass fraction for those species were therefore derived from other published syntheses of herbivore diet composition (Abraham et al., 2022; Gagnon & Chew, 2000).

For all 57 species present across the 23 communities in our analyses, we extracted species‐level body mass data from EltonTraits, a dataset compiled from primary literature containing species‐level trait data and foraging attributes for all bird and mammal species (Wilman et al., 2014). Site‐specific body mass data do not exist for most communities, which prevented us from evaluating the impact of spatial variation in herbivore body mass on grass dependence in this study. Note that body mass values were log‐transformed for all analyses.

### 2.3 Environmental covariates

To evaluate whether environmental drivers altered the relationship between grass dependence and body mass across herbivore communities, we downloaded shapefile polygons for the protected areas corresponding to each herbivore community in our analysis from the World Database on Protected Areas (WDPA); though some of the communities spanned multiple protected areas (Cerling et al., 2015), the majority of samples for each community were collected from within a single protected area, which was the protected area we used to extract environmental covariates (see Appendix S1 for the primary protected area corresponding to each community).

Using climatic data layers downloaded from WorldClim 2.0 at a spatial resolution of 30′ (Fick & Hijmans, 2017), we then calculated mean annual rainfall (‘MARain’), mean rainfall seasonality (‘RainS’), mean annual temperature (‘MATemp’), and mean temperature seasonality (‘TempS’) for each community. Rainfall seasonality data from WorldClim are quantified in terms of the coefficient of variation (standard deviation divided by the mean) in precipitation throughout the year, whereas temperature seasonality values are quantified as the standard deviation in temperature across the seasonal cycle; they are measured differently because temperature can take on zero or negative values, whereas precipitation cannot (Fick & Hijmans, 2017). WorldClim data layers were generated by aggregating data spanning 1970–2000 from 9000 to 60,000 weather stations and interpolating weather station data with covariates including elevation, distance to coast, and satellite‐derived land surface temperatures to produce high‐resolution global layers (Fick & Hijmans, 2017). Climatic data used in this study therefore reflect long‐term (e.g., decadal) climate averages experienced by each herbivore community.

We also wanted to evaluate whether differences in grass availability might underlie variation in the relationship between grass dependence and body mass across communities. Though datasets of grass biomass across regional scales are largely lacking (but see Sala et al., 2012), grass biomass should vary inversely with woody cover to some degree (though not perfectly; see Sokolowski et al., 2023). We therefore extracted estimates of woody cover for each community (‘Woody_cover’) from a data layer of fractional woody cover for sub‐Saharan Africa (Venter et al., 2018). This data layer was generated via a Random Forest regression model using Landsat surface reflectance data available for Africa (1986–2016) from the USGS Earth Resources Observation and Science archive (Venter et al., 2018). The Random Forest model was trained on high‐resolution images derived from Google Earth (4000 randomly scattered 30 × 30 m sampling quadrats aligned with the Landsat pixel grid) for which fractional woody cover was then manually classified (by identifying woody plant canopies using texture, color, and canopy shadows; Venter et al., 2018). Then, using the Landsat data to extrapolate from these 4000 manually classified quadrats, the Random Forest model was used to predict fractional woody plant cover (defined as fractional woody cover within each 30 × 30 m pixel of the Landsat grid) for the whole of sub‐Saharan Africa (Venter et al., 2018). Woody cover data employed here therefore reflect the areal extent of trees and shrubs relative to grasses, herbaceous vegetation, and unvegetated landscape for each herbivore community (Sokolowski et al., 2023; Venter et al., 2018).

### 2.4 Data analysis

Data were analyzed in R v3.6.1 (R Core Team, 2020). Because dietary grass fraction values were bounded between 0 and 1, we modeled the relationship between dietary grass values and body mass within each community using beta‐regression, which prevents fitting values beyond 0 and 1, using the R package ‘betareg’ (Cribari‐Neto & Zeileis, 2010). Beta‐regression does not allow input data to take on values of 0 or 1 however, so 0s were converted to .001 and 1s to .999. To evaluate the impact of incomplete community data on results, we built two separate beta‐regression models: one model included only the local diet estimates but included data from all 23 communities, and a second model included both local and regional diet estimates for the 15 communities for which we had complete community data. To avoid type I errors resulting from multiple hypothesis testing, relationships between body mass and grass dependence were modeled simultaneously for all communities: we built a single model of dietary grass fraction and included community identity and log‐transformed body mass as interacting predictors of dietary grass fraction, which yielded separate slope estimates (and associated errors) for each community. Model fit statistics and coefficient estimates therefore account for multiple comparisons (see Appendix S2). To incorporate local within‐species diet variation and propagate uncertainty across analyses, species‐level dietary grass fraction values were weighted by their corresponding standard deviations in the beta‐regression models.

Because some of the most grass‐specialist herbivores are of intermediate body size (~50 to 400 kg) (Codron et al., 2007; Gagnon & Chew, 2000; Hempson, Archibald, et al., 2015; Jarman, 1974), it is plausible that the relationship between body size and grass dependence might be parabolic, with grass intake peaking at intermediate body sizes. To evaluate whether a first‐order or second‐order relationship between body mass and grass dependence better accorded with available data, we constructed beta‐regression models with linear and parabolic relationships and compared both model‐corrected Akaike information criterion (AIC_c_) and Root Mean Square Error (RMSE) values between the models. For both AIC_c_ and RMSE, lower values are indicative of a better model fit. When only local diet data were considered, a linear relationship (AIC_c_ = −34.486, RMSE = 0.320) fit the data better than did a parabolic relationship (AIC_c_ = −30.106, RMSE = 0.325); likewise, when communities with complete data were considered, a linear relationship (AIC_c_ = −82.142, RMSE = 0.297) again better fit the data than did a parabolic relationship (AIC_c_ = −71.680, RMSE = 0.299). We thus assumed a first‐order relationship between body size and grass dependence for all subsequent analyses.

**FIGURE 2:**
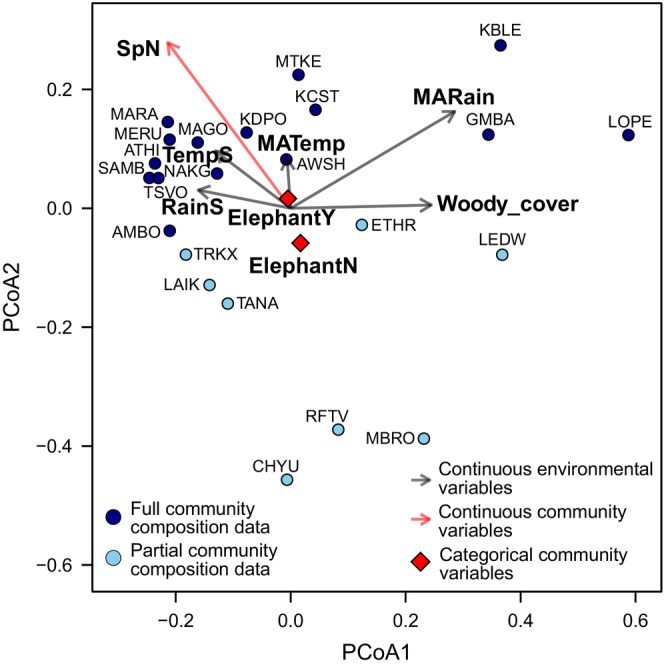
Jaccard‐based principle coordinate analysis (PCoA) of herbivore species composition within central and eastern African herbivore communities: AMBO, Amboseli; ATHI, Athi Plains and Nairobi; AWSH, Awash; CHYU, Chyulu Hills; ETHR, Ethiopian Rift Lakes; GMBA, Garamba; KBLE, Kibale; KCST, Kenya Coast; KDPO, Kidepo; LAIK, Laikipia; LEDW, Lake Edward; LOPE, Lopé; MAGO, Omo/Mago; MARA, Maasai Mara–Serengeti; MBRO, Lake Mburo; MERU, Meru National Park; MTKE, Mount Kenya; NAKG, Nakuru; RFTV, Kenya Rift Valley; SAMB, Samburu; TANA, Tana River; TRKX, Turkana; TSVO, Tsavo. Communities represented by *dark blue points* were present in both stable isotope and community composition datasets and therefore reflect the complete herbivore community. Communities represented by *light blue points* were absent from our community composition dataset, and therefore herbivore community composition data may be incomplete. Relevant variables are plotted as well (depicted in bold), with *gray arrows* corresponding to continuous environmental variables and *red arrows* corresponding to continuous community variables. *Red diamonds* correspond to categorical community variables.

Aspects of the herbivore community might also contribute to variation in the strength of the body mass‐dietary grass fraction relationship, either in concert with or independently of the environmental variables described above. To visualize variation in herbivore community composition and to evaluate the impacts of community composition on the relationship between body mass and grass dependence, we used Jaccard‐based principle coordinates analysis (PCoA) to ordinate community composition (Figure 2) in the R package ‘vegan’ (Dixon, 2003). Also using the R package ‘vegan’ (Dixon, 2003), we plotted environmental co‐variates on our ordination to assess whether environmental predictors were colinear with community composition variables (see also Figure S1). Indeed, woody cover (‘Woody_cover’) and mean annual rainfall (‘MARain’) loaded strongly on axis 1 of our PCoA (Figure 2; Figure S1). To account for variation in community composition not explained by environmental variables, we extracted the values for each herbivore community along the first two PCoA axes, axis 1 (‘PCoA1’) and axis 2 (‘PCoA2’), as well as the herbivore species richness (‘SpN’) and mean grass dependence (‘mean_C_4_’) of each community, for inclusion as predictors of the body mass‐grass dependence relationship. We included species richness (‘SpN’) as a predictor because a strongly positive relationship between body mass and dietary grass fraction might only manifest in species‐rich communities where herbivores are sufficiently specialized in different plant functional types (Pansu et al., 2022; Pringle et al., 2023). Similarly, we included community‐averaged grass dependence (‘mean_C_4_’) because a positive relationship might only arise in communities where grass is readily available and herbivores consume significant amounts of grass (Bhat et al., 2020). We also categorized communities by whether or not elephants (*Loxodonta* spp.) were present and included this (‘Elephant’) as a categorical predictor of slope. Our rationale for treating elephants uniquely was that elephants are the largest extant herbivore in these communities and often dominate savanna herbivore communities in terms of biomass (Hempson, Archibald, et al., 2015); elephants play an outsized role in structuring vegetation communities (Abraham et al., 2021; Cardoso et al., 2020); elephants are known to induce fear effects in co‐occurring herbivores (Fletcher et al., 2023), altering the foraging behavior of heterospecifics (Landman et al., 2013; Valeix et al., 2007); and finally, though modern‐day elephants incorporate substantial amounts of browse in their diets, elephantids were predominately grazers for much of their fossil history (up until ~1 Ma), such that their current predominately browsing habit is not entirely representative (Cerling et al., 1999; Lister, 2013).

To determine which of these predictors best‐explained variation in the relationship between body mass and grass dependence, we constructed a series of linear models including all possible combinations of predictor variables and compared AIC_c_ values to assess model fit using the R package ‘MuMIn’ (Bartoń, 2019). We did this using two different datasets, one with the slope estimates from the beta‐regression model that included the local diet data for all 23 communities (‘Local diet data only’) and another with the slope estimates from the beta‐regression model that included both local and regional diet estimates but only the 15 communities for which we had complete community data (‘Full community diet data’) (see Tables S3–S6). The relationship between body mass and dietary grass fraction was weighted by the standard error on the slope, as above, to propagate uncertainty in the slope estimates across analyses. Because communities were distributed unevenly in space (Figure 1), we accounted for potential spatial autocorrelation by including various spatial covariance structures in global models and evaluating whether they improved model fit (Table S3). The spatial covariance structure that resulted in the lowest AIC_c_ value was then included in all models within a given model set.

Due to the collinearity, we observed above between herbivore community and environmental variables (Figure S1), we evaluated three separate model sets for both of these datasets (Table S3): one with only environmental predictors (i.e., ‘MARain’, ‘RainS’, ‘MATemp’, ‘TempS’, and ‘Woody_cover’), one with only herbivore community predictors (i.e., ‘PCoA1’, ‘PCoA2’, ‘SpN’, ‘mean_C_4_’, and ‘Elephant’), and one with both environmental and community predictors (see Table S3). In the two model sets including environmental predictors, we also included the second‐order polynomial transformations of ‘MARain’ and ‘Woody_cover’ as predictors (Table S3), to evaluate whether the slope of the body mass‐grass dependence relationship might be most extreme at intermediate rainfall and/or woody cover (see Olff et al., 2002). As a further precaution against overfitting, we excluded from model selection any models with predictors that covaried significantly with one another, such that only one of any pair of correlated variables (defined here as significantly correlated variables with correlation coefficients *r* > .5) was included in any given model (Figure S1). We then compared model sets to determine which predictors arose regularly in plausible models and had consistent directional effects across models (Tables S4–S6). We considered all models with ΔAIC_c_ < 2 as ‘equally plausible’ models (Anderson & Burnham, 2002).

We repeated the above modeling procedure with community‐averaged dietary grass fraction as the response variable (instead of the slope of the grass dependence‐body mass relationship) to evaluate predictors of variation in the average reliance of the herbivore communities on grass (Tables S7–S10).

For all of the above analyses, we also ran models that excluded pure browsers (i.e., herbivores with dietary grass fraction >0), to assess if results were consistent when considering only herbivores that consume some amount of grass (see Tables S3–S10). Results presented below were qualitatively the same between models including all herbivores and only herbivores consuming some grass unless otherwise noted.

Lastly, to explicitly interrogate the role of intraspecific diet shifts in contributing to community‐level patterns, we modeled intraspecific diet variation for seven widespread herbivore species (see Table S11): plains zebra (*Equus quagga*), giraffe (*Giraffa camelopardalis*), common hippopotamus (*Hippopotamus amphibius*), waterbuck (*Kobus ellipsiprymnus*), savanna elephant (*Loxodonta africana*), common warthog (*Phacochoerus africanus*), and Cape buffalo (*Syncerus caffer*). We restricted our analyses to these seven species because local diet data for these species were available from more than half of the communities (*N* ≥ 12). We replicated the modeling approach employed above for each species: we constructed species‐specific linear models of dietary grass fraction that included the same predictor variables as above and compared AIC_c_ values to assess model fit. As above, species‐level dietary grass fraction values were weighted by their corresponding standard deviations in all models, to account for local intraspecies diet variation and propagate uncertainty. We again accounted for potential spatial autocorrelation by evaluating various spatial covariance structures and selecting the spatial covariance structure that resulted in the lowest AIC_c_ value for inclusion in the global model (Table S11). As above, we evaluated three separate model sets (Table S11): one with only environmental predictors (Table S12), one with only herbivore community predictors (Table S13), and one with both environmental and community predictors (Table S14). Note that, in contrast to the community‐level analyses, which we performed both on local diet data only as well as including regional diet averages for those species lacking local data, we only conducted these analyses using local diet data. Likewise, because all seven species ate some amount of grass in every community, it was not necessary to evaluate models that excluded pure browsers, as we had done for community‐level analyses.

## 3 RESULTS

**FIGURE 3:**
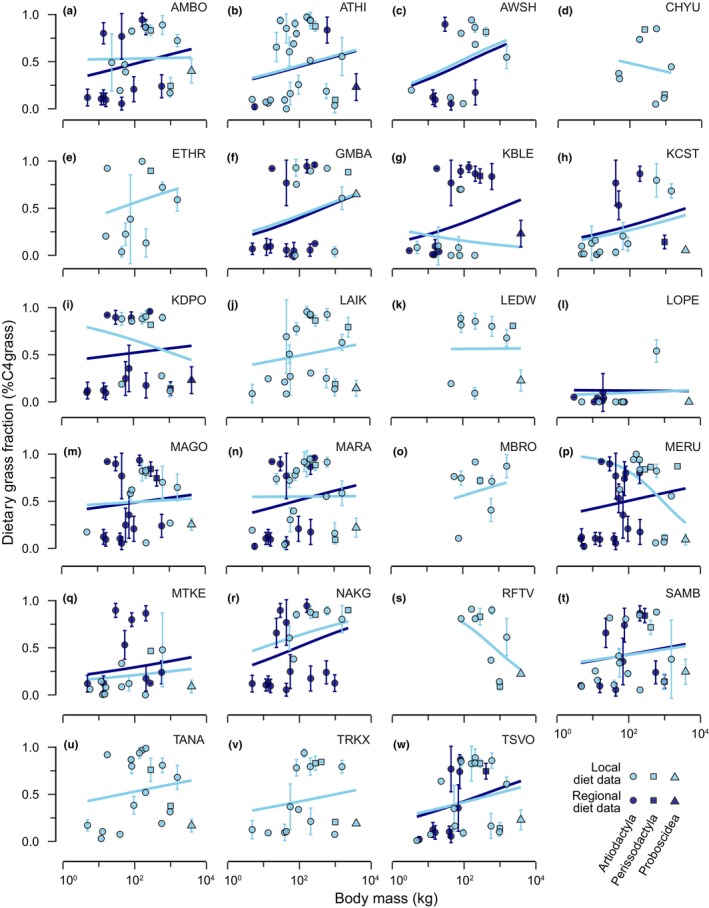
The relationship between body mass and dietary grass fraction within African large mammalian herbivore communities: (a) AMBO, Amboseli; (b) ATHI, Athi Plains and Nairobi; (c) AWSH, Awash; (d) CHYU, Chyulu Hills; (e) ETHR, Ethiopian Rift Lakes; (f) GMBA, Garamba; (g) KBLE, Kibale; (h) KCST, Kenya Coast; (i) KDPO, Kidepo; (j) LAIK, Laikipia; (k) LEDW, Lake Edward; (l) LOPE, Lopé; (m) MAGO, Omo/Mago; (n) MARA, Maasai Mara–Serengeti; (o) MBRO, Lake Mburo; (p) MERU, Meru National Park; (q) MTKE, Mount Kenya; (r) NAKG, Nakuru; (s) RFTV, Kenya Rift Valley; (t) SAMB, Samburu; (u) TANA, Tana River; (v) TRKX, Turkana; (w) TSVO, Tsavo. The slope of the relationship between body mass and dietary grass fraction was positive for most herbivore communities, especially when data on the entire herbivore community were considered, but the magnitude of the slope varied across communities. Point shapes correspond to mammalian herbivore orders, point colors correspond to whether diet data were local (*light blue points*) or regional (average dietary grass fraction for a species across all communities; *dark blue points*). *Light blue lines* reflect the relationship for only those species for which local diet data were available; *dark blue lines* reflect the relationship for the entire community (including species for which only regional averages were available).

When only local diet data were considered (beta‐regression; pseudo‐*R* ^2^ = .362, *N* = 288, logLik = 73.643, df = 47, AIC_c_ = −34.486), 18 out of 23 communities exhibited positive relationships between body mass and dietary grass fraction (Figure 3), though the two communities that exhibited robustly directional slopes (*p* < .1), Meru National Park (MERU) and Kenya Rift Valley (RFTV), had negative relationships (Table S1). When data on the full herbivore community were considered, incorporating both local diet data and regional averages for taxa missing local data (beta‐regression; pseudo‐*R* ^2^ = .255, *N* = 443, logLik = 93.782, df = 47, AIC_c_ = −82.142), 14 out of the 15 communities for which herbivore community data were available exhibited positive relationships (Figure 3), and for four communities the relationship between body mass and dietary grass fraction was robustly positive (*p* < .1; Table S1). These patterns were qualitatively consistent when only herbivores consuming some amount of grass (dietary grass fraction >0) were considered (Table S2), with 17 out of 22 communities exhibiting positive relationships between body mass and dietary grass fraction when local diet data only were considered (beta‐regression; pseudo‐*R* ^2^ = .267, *N* = 275, logLik = 39.046, df = 45, AIC_c_ = 29.986) (Table S2). When data on the full herbivore community were considered (beta‐regression; pseudo‐*R* ^2^ = .184, *N* = 428, logLik = 51.298, df = 47, AIC_c_ = 3.278), all 15 communities exhibited positive relationships, and four robustly so (*p* < .1; Table S2). Though the relationship between body mass and dietary grass fraction was positive for most communities, larger herbivores did not universally eat more grass within these communities. Instead, the range of diets seemed to increase with body size: small herbivores (<10 kg) were almost universally browsers, whereas larger herbivores ranged from specialist browsers to specialist grazers, such that larger‐bodied herbivores did eat more grass on average (Figure 3). The positive relationship between body size and dietary grass fraction within these communities thus reflects the fact that variability in dietary grass fraction increases with body size.

**FIGURE 4:**
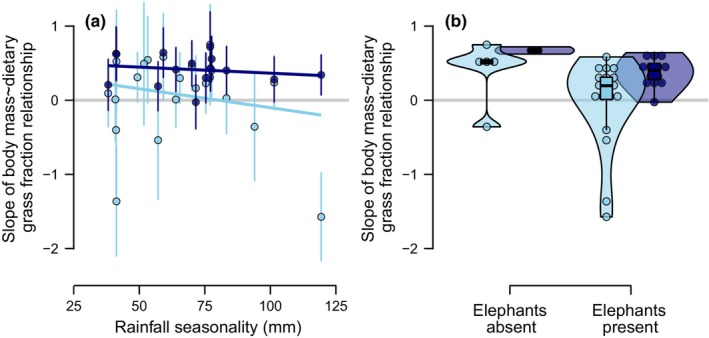
Determinants of the slope of the relationship between body mass and dietary grass fraction for large mammalian herbivore communities. Across data subsets, the slope of the relationship between body mass and dietary grass fraction declined with increasing rainfall seasonality and where elephants were present in the community. Point colors correspond to whether only local diet data were considered (*light blue points*) or whether regional diet data were also included (for systems where full community composition data were available; *dark blue points*). *Light blue lines* reflect the relationships across all 23 communities but considering only local diet data; *dark blue lines* reflect the relationships across communities considering only those 15 communities with full community composition data (and therefore both local and regional diet data).

Though the slope of the relationship between body mass and dietary grass fraction was positive for the majority of communities, the magnitude of the slope did vary across communities even so, and rainfall seasonality proved predictive of that variation (Figure 4a): increasing rainfall seasonality resulted in less positive slopes, especially when local diet data only were considered (Figure 4a; Table S4). In other words, grass intake increased less predictably with body size where rainfall was more seasonally variable. When complete community data were considered, however, rainfall seasonality had less predictive power for explaining variation in slope (Figure 4a; Table S4).

Whether or not elephants were present in a community was highly predictive of variation in the relationship between body mass and dietary grass fraction across communities (Figure 4b; Tables S5 and S6), more so even than rainfall seasonality. The presence of elephants consistently decreased the slope of the relationship, indicating that grass dependence did not increase as strongly with body size in communities where elephants were present. The negative effect of elephant presence on the slope of the body mass‐dietary grass fraction relationship was remarkably consistent across datasets: elephant presence featured within the plausible model sets for all datasets (Tables S4 and S6), and in every plausible model when only local diet data for herbivores consuming some amount of grass (dietary grass fraction >0) were considered (Table S6). Furthermore, for 16 out of the 18 communities in which elephants were present, the slope of the relationship between body mass and dietary grass fraction became substantially more positive when elephants were excluded (Figure 3). Though other herbivore community composition variables did feature variously in plausible models, their effects were not consistent across datasets, indicating that the strength of the relationship between body mass and dietary grass fraction does not otherwise strongly depend on the composition of the herbivore community.

**FIGURE 5:**
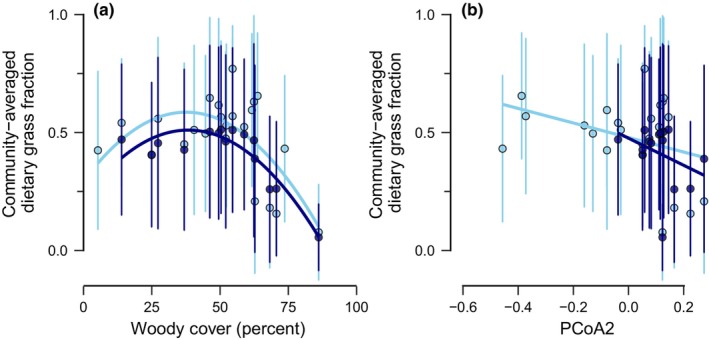
Determinants of community‐averaged dietary grass fraction for large mammalian herbivore communities. Across all data subsets, community‐averaged dietary grass fraction peaked at intermediate woody cover and declined with a community's position along PCoA axis 2. Point colors correspond to whether only local diet data were considered (*light blue points*) or whether regional diet data were also included (for systems where full community composition data were available; *dark blue points*). *Light blue lines* reflect the relationships across all 23 communities but considering only local diet data; *dark blue lines* reflect the relationships across communities considering only those 15 communities with full community composition data (and therefore both local and regional diet data).

We also evaluated what underlay variation in the average reliance of these herbivore communities on grass. The average proportion of grass consumed by herbivore community members was strongly influenced by woody cover, with community‐averaged dietary grass fraction peaking at intermediate woody cover (Figure 5a; Tables S9 and S10). This pattern held regardless of whether only local diet data or complete community data were considered (Figure 5a; Tables S9 and S10). The species composition of herbivore communities was also highly predictive of community‐averaged dietary grass fraction (Tables S8 and S10). The average grass dependence of a community was strongly negatively correlated with that community's position along PCoA axis 2 (Figure 5b; Table S8), indicating that the average grass dependence of a community depends strongly upon the species present in that community.

**FIGURE 6:**
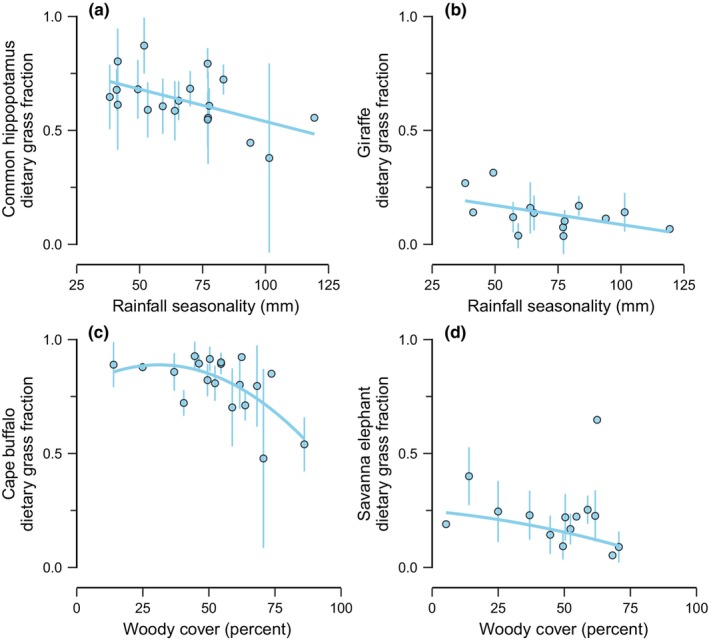
Contribution of intraspecific diet variation to community‐level patterns. For both (a) common hippopotamus (*Hippopotamus amphibius*) and (b) giraffe (*Giraffa camelopardalis*), dietary grass fraction declined with increasing rainfall seasonality, contributing to the dependence of the slope of the body mass‐dietary grass fraction relationship on rainfall seasonality. Similarly, (c) Cape buffalo (*Syncerus caffer*) and (d) savanna elephant (*Loxodonta africana*) dietary grass fraction were parabolically related to woody cover, paralleling the parabolic relationship between community‐averaged dietary grass fraction and woody cover.

Last, we evaluated how intraspecific dietary variation for widely distributed herbivore taxa contributed to these community‐level patterns. Species differed markedly in how predictable their diets were overall and the factors that best predicted intraspecific diet variation (Tables S11–S14). Rainfall seasonality was a key predictor of intraspecific diet variation for the common hippopotamus (*H. amphibius*) and giraffe (*G. camelopardalis*) (Tables S13 and S14): the grass dependence of both species declined with increasing rainfall seasonality (Figure 6a,b), much as the slope of the body mass‐dietary grass fraction relationship declined with increasing rainfall (Figure 4a). Similarly, woody cover was a key predictor of intraspecific diet variation for Cape buffalo (*S. caffer*) and savanna elephant (*L. africana*) (Tables S13 and S14): grass dependence for both species was parabolically related to woody cover (Figure 6c,d), paralleling the parabolic relationship between community‐averaged dietary grass fraction and woody cover (Figure 5a).

Herbivore community composition variables had limited predictive power for explaining spatial variation in dietary grass fraction for these seven herbivore species. Across all the models of intraspecific diet variation that included herbivore community composition variables only, the null model was always the model with the lowest AIC_c_, except in the case of Cape buffalo (*S. caffer*), where dietary grass fraction declined with the location of the broader herbivore community along PCoA axis 1 (Table S12). However, the predictive power of PCoA1 for explaining buffalo diet variation appeared to be a consequence of the fact that woody cover loaded strongly on PCoA axis 1 (Figure 2): woody cover was the best predictor of intraspecific dietary variation when environmental variables were included, both when only environmental variables were considered (Table S13) and when environmental and community variables were considered together (Table S14). As such, dietary grass fraction for these seven herbivore species is largely decoupled from characteristics of the herbivore communities within which they are situated.

## 4 DISCUSSION

Here we found that (1) dietary grass fraction increased with body size for the majority of African herbivore communities we considered (Figure 3), especially when complete community data were considered. That said, the magnitude of this relationship varied, and (2) rainfall seasonality underlay some of the variation in the strength of the relationship (Figure 4): grass dependence increased less strongly with body size where rainfall seasonality was greater. Similarly, (3) dietary grass fraction also did not increase as strongly with body size in communities where elephants were present (Figure 4). Furthermore, (4) the reliance of herbivore communities on grass was strongly tied to woody cover, peaking at intermediate woody cover, as well as the species composition of the community (Figure 5). Finally, we found that (5) intraspecific diet shifts contributed to these community‐level patterns: common hippopotamus (*H. amphibius*) and giraffe (*G. camelopardalis*) grass dependence decreased with increasing rainfall seasonality, whereas Cape buffalo (*S. caffer*) and savanna elephant (*L. africana*) grass dependence were parabolically related to woody cover (Figure 6).

The positive relationship between body size and grass consumption documented within certain herbivore communities and across herbivore species (Abraham et al., 2022; Bell, 1971; Cerling et al., 2003; Gagnon & Chew, 2000; Jarman, 1974) largely held at the community level: within the majority of communities analyzed here, dietary grass fraction increased with body size on average. This was particularly true when complete community data were analyzed, emphasizing the importance of having complete data when drawing conclusions about community‐level patterns. Furthermore, we found that herbivore community variables, such as herbivore species richness, community‐averaged grass dependence, and species composition, had little power for explaining variation in the relationship between body mass and grass dependence. The positive relationship between grass dependence and body size is therefore not unique to particular community configurations, but rather a general principle of savanna herbivore community assembly. That the positive relationship between body mass and grass dependence manifests across a diversity of herbivore communities is encouraging, as it suggests that some general (and scale‐independent) principles do underlie herbivore foraging behavior (Abraham et al., 2022; Olff et al., 2002) and therefore also their impacts on plants (Karp et al., in revision; Staver et al., 2021). Still, the mechanism behind this relationship remains elusive, and further research should attempt to disentangle whether high grass dependence is possible for large herbivores due to the comparatively homogeneous distribution of grasses (Bhat et al., 2020; Staver, Asner, et al., 2019) or shallower nutrient gradients across grass tissues (Güsewell, 2004; Potter & Pringle, 2023; Reich & Oleksyn, 2004).

One herbivore community variable that did explain variation in the magnitude of the slope was the presence of elephants. Elephant presence consistently decreased the slope of the relationship between grass dependence and body size. There are two pathways by which elephant presence might negatively impact the slope. First, elephants might induce diet shifts in other herbivores, either via resource competition or via fear effects (Fletcher et al., 2023; Landman et al., 2013; Valeix et al., 2007), causing heterospecifics to eat less grass and thereby deflating the slope. While such competitive and fear effects are well‐documented for elephants (Fletcher et al., 2023; Landman et al., 2013; Valeix et al., 2007), our results suggest that these effects do not alter the amount of grass that other herbivore species consume: elephant presence was not included in any species‐specific models of intraspecific dietary variation across communities. The alternative pathway by which elephant presence might drag down the slope is simply by their being so large and consuming relatively little grass. Indeed, excluding elephants from our analyses made the slope substantially more positive for 16 out of the 18 communities in which elephants were present (Figure 3). As such, elephants appear to eat substantially less grass than might be expected from their body size alone, a pattern that has been noted elsewhere (Cerling et al., 1999; Lister, 2013): despite the fact that they are the largest extant herbivores in these communities (Pansu et al., 2022; Rowan et al., 2020) and possess morphological adaptations to grazing, such as high‐crowned teeth (Cerling et al., 1999; Lister, 2013), elephants in eastern and central Africa (Cerling et al., 1999, 2015; Robinson et al., 2021) and beyond (Abraham et al., 2019; Pansu et al., 2022) are predominantly browsers. Furthermore, though modern African elephants mainly browse, elephantids were grazers for much of their evolutionary history, such that their current browsing habit may not be broadly representative: grasses dominated the diets of *Loxodonta* from 5 to 1 Ma (Cerling et al., 1999). Why modern elephants eat so little grass remains a mystery (Cerling et al., 1999) but implies that some change in their resource environment has occurred within the last 1 million years; elephants are one of the few taxa whose diets vary predictably with woody cover (Figure 6; see also Robinson et al., 2021), indicating that they may be more sensitive to changes in resource availability relative to other herbivores (see also Abraham et al., 2019, 2021). Regardless, the centrality of elephants to our results underscores the unique position that elephants occupy within herbivore communities: not only do elephants forage differently than other herbivores (Abraham et al., 2021; Robinson et al., 2021), they consume immense amounts of plant biomass, often constitute the majority of herbivore biomass within herbivore communities, and play a key role in ecosystem construction overall (Abraham et al., 2021; Cardoso et al., 2020; Hempson, Archibald, et al., 2015).

Rainfall seasonality also explained some of the variations in the relationship between body mass and grass dependence, with increasing rainfall seasonality leading to less strongly positive slopes. Rainfall seasonality likely modifies herbivore foraging behavior by altering plant availability and quality. Grass is particularly responsive to rainfall (Sala et al., 2012; Scanlon et al., 2005; Staver, Wigley‐Coetsee, et al., 2019), such that severe dry seasons may result in seasonally sparse (Abraham et al., 2022; Hempson, Illius, et al., 2015) or especially low quality (Hempson et al., 2019; Reich & Oleksyn, 2004) grass forage, undermining any foraging advantage that specializing on grass presents (Bhat et al., 2020). Indeed, we found that some species did consume less grass where rainfall was more seasonal. The two species for which this was the case, the common hippopotamus and giraffe, are also two of the largest species in our analyses. Thus, highly seasonal rainfall seems to erode the foraging advantage of consuming grass, particularly for large herbivores. Still, whether this is driven by changes in grass availability (Abraham et al., 2022; Bhat et al., 2020; Hempson, Illius, et al., 2015) or relative quality (Hempson et al., 2019; Reich & Oleksyn, 2004) remains unclear. Regardless, these results indicate that herbivore diets result from the dynamic interplay between herbivore physiology and environmental context (Abraham et al., 2019; Hopcraft et al., 2010; Veldhuis et al., 2019), with cascading consequences for community assembly.

Environmental context also altered the average grass dependence of the herbivore community as a whole. Dietary grass fraction peaked at intermediate woody cover, somewhat surprisingly: we expected dietary grass fraction to monotonically decrease with woody cover, as herbivores should presumably eat more browse where it is more abundant (Pansu et al., 2022; Robinson et al., 2021). However, grass biomass might not be negatively related to woody cover, which would result in non‐linearities in the relative abundance of grasses with increasing woody cover; savannas have a continuous grassy understory (Riginos et al., 2009; Sala et al., 2012; Staver, 2018), and both woody cover and grass biomass increase with rainfall (Sala et al., 2012; Staver et al., 2011; Venter et al., 2018), such that grass biomass may be largely invariant with, or even increase with, tree cover up to a certain threshold. Also, savanna trees have been observed to facilitate grasses, locally increasing grass biomass (Moustakas et al., 2013; Riginos et al., 2009), such that systems with intermediate woody cover may have higher grass biomass than those without trees. Finally, not all communities with sparse woody cover are necessarily grasslands—many of the communities in our analyses, especially those with low woody cover, are arid or semi‐arid systems (MARain <800 mm) that likely have some amount of unvegetated land, which would further decouple grass biomass from woody cover (Sokolowski et al., 2023). Unfortunately, fine‐grained, temporally explicit datasets of local grass biomass across regional scales are lacking (but see Sala et al., 2012), which prevents us from explicitly considering the role of grass availability in mediating the effects of woody cover on herbivore community grass dependence here. An additional possibility is that systems with intermediate woody cover might have a greater potential diversity of dietary niches due to greater resource diversity therein (Olff et al., 2002; Tilman, 1982); in our analyses, sites with intermediate woody cover also had intermediate MARain values (Figure S1; see also Sokolowski et al., 2023), which is where theory predicts forage quality and quantity to be mutually maximized (Olff et al., 2002). Thus, grass may only be sufficiently palatable and abundant at intermediate woody cover, such that specialist grazers might be uniquely able to persist in communities with some amount of woody cover (Hempson, Archibald, et al., 2015), inflating the average grass dependence of those communities with intermediate woody cover. In support of this latter possibility, we note that both overall herbivore biomass densities and grazer biomass densities specifically are observed to peak at intermediate woody cover (Hempson, Archibald, et al., 2015; Staver et al., 2021). To determine how grass availability alters the grass dependence of individual community members and the community as a whole, we require better data on grass availability across continental scales (Sala et al., 2012): with ongoing advances in remote sensing technologies (Cavender‐Bares et al., 2022; Scanlon et al., 2005), this will become increasingly possible. Regardless, these patterns suggest that herbivore impacts on the grass layer should peak at intermediate woody cover (see also Karp et al., in revision; Staver et al., 2021).

Finally, we found that both species turnover and intraspecific diet shifts contributed to variation in community‐averaged dietary grass fraction. Grass fraction was anti‐correlated with PCoA axis 2, such that PCoA axis 2 appears to differentiate those herbivore communities with both grazing and browsing species from communities dominated mainly by browsers. That species composition contributes to how dependent a community is on grass underscores the fact that herbivore species in the tropics differ markedly in their dietary niches: species range from pure browsers to near‐obligate grazers (Abraham et al., 2022; Gagnon & Chew, 2000; Pansu et al., 2022). Still, variation in community‐level grass dependence was not entirely driven by species turnover between communities. Paralleling the parabolic relationship between community‐averaged grass dependence and woody cover, we found that Cape buffalo and savanna elephant diets were likewise parabolically related to woody cover (see also Robinson et al., 2021). Therefore, while the dietary niches of species present in a community play a large role in mediating community‐averaged grass dependence, spatial shifts in herbivore diets also appear to contribute. These findings emphasize that intraspecific dietary variation can have community‐level consequences (Pansu et al., 2022; Pringle et al., 2023) and that local diet data are therefore essential for understanding community processes and accurately estimating local herbivore impacts (Pringle et al., 2023).

In summary, we found that herbivore diets were somewhat predictable from body size, even at the scale of particular herbivore communities. That dietary grass fraction, or rather the variability of possible diets, increases with body size therefore appears to be a general principle of community assembly across savanna herbivore communities. Consequently, the body size distribution of herbivore communities defines the magnitude of herbivore impacts on the grass and tree layers, especially within less rainfall‐seasonal systems where the relationship between body size and grass dependence is strongest. Still, the mechanism behind this positive body size‐grass dependence relationship remains unclear. Future research should leverage variation in the relationship between body size and grass dependence such as we have documented here to identify the mechanism by which this relationship manifests, explicitly linking the strength of the body mass–grass dependence relationship to differences in resource quality and resource distributions across large mammalian herbivore communities.

## AUTHOR CONTRIBUTIONS

**Joel O. Abraham:** Conceptualization (equal); data curation (equal); formal analysis (lead); investigation (lead); methodology (lead); project administration (lead); visualization (lead); writing – original draft (lead); writing – review and editing (lead). **John Rowan:** Conceptualization (equal); data curation (supporting); investigation (supporting); methodology (supporting); project administration (supporting); writing – review and editing (supporting). **Kaedan O'Brien:** Data curation (supporting); methodology (supporting); writing – review and editing (supporting). **Kathryn G. Sokolowski:** Investigation (supporting); writing – review and editing (supporting). **J. Tyler Faith:** Conceptualization (equal); data curation (lead); formal analysis (supporting); investigation (equal); methodology (supporting); project administration (supporting); supervision (equal); writing – review and editing (supporting).

## Supporting information


Appendix S1

Appendix S2

## Data Availability

Data are available in the supplementary data file associated with this publication and from the Dryad Data Repository (https://doi.org/10.5061/dryad.rv15dv4fs).
