# Supplementary material for: Environmental context shapes the relationship between grass consumption and body size in African herbivore communities
Source: Ecol Evol. 2024 Feb 15;14(2):e11050. doi: 10.1002/ece3.11050 (PMC10867881; doi:10.1002/ece3.11050)
Supplement: Supplementary file 2 — Appendix S2 [file ECE3-14-e11050-s001.docx]

**SUPPLEMENTARY MATERIALS**

**Table S1.** Community-specific estimates of the slope of the relationship between body mass and dietary grass fraction for large mammalian herbivore communities across central and eastern African communities**,** including all herbivores. Slopes correspond to those plotted in figure 3. Model fit statistics and coefficient estimates account for multiple comparisons.

| Comm-unity | Local diet data | | | | Full community diet data | | | |
| --- | --- | --- | --- | --- | --- | --- | --- | --- |
|  | Slope | Std. Error | *Z*-value | *P*-value | Slope | Std. Error | *Z*-value | *P*-value |
| AMBO | 0.028 | 0.487 | 0.058 | 0.954 | 0.401 | 0.330 | 1.217 | 0.223 |
| ATHI | 0.426 | 0.367 | 1.159 | 0.246 | 0.428 | 0.312 | 1.372 | 0.170 |
| AWSH | 0.748 | 0.546 | 1.371 | 0.170 | 0.720 | 0.476 | 1.512 | 0.131 |
| CHYU | -0.358 | 0.732 | -0.489 | 0.625 | NA | NA | NA | NA |
| ETHR | 0.544 | 0.588 | 0.924 | 0.355 | NA | NA | NA | NA |
| GMBA | 0.583 | 0.592 | 0.986 | 0.324 | 0.641 | 0.339 | 1.890 | 0.059 |
| KBLE | -0.402 | 0.869 | -0.462 | 0.644 | 0.628 | 0.368 | 1.706 | 0.088 |
| KCST | 0.459 | 0.333 | 1.378 | 0.168 | 0.496 | 0.312 | 1.588 | 0.112 |
| KDPO | -0.539 | 0.807 | -0.669 | 0.504 | 0.189 | 0.336 | 0.561 | 0.575 |
| LAIK | 0.297 | 0.346 | 0.860 | 0.390 | NA | NA | NA | NA |
| LEDW | 0.011 | 0.573 | 0.020 | 0.984 | NA | NA | NA | NA |
| LOPE | 0.163 | 0.440 | 0.369 | 0.712 | -0.026 | 0.366 | -0.070 | 0.944 |
| MAGO | 0.093 | 0.458 | 0.202 | 0.840 | 0.207 | 0.349 | 0.593 | 0.553 |
| MARA | 0.010 | 0.381 | 0.026 | 0.979 | 0.413 | 0.303 | 1.364 | 0.173 |
| MBRO | 0.493 | 0.833 | 0.592 | 0.554 | NA | NA | NA | NA |
| MERU | -1.573 | 0.596 | -2.637 | 0.008 | 0.341 | 0.276 | 1.236 | 0.216 |
| MTKE | 0.228 | 0.410 | 0.558 | 0.577 | 0.300 | 0.342 | 0.879 | 0.380 |
| NAKG | 0.523 | 0.696 | 0.751 | 0.453 | 0.622 | 0.359 | 1.733 | 0.083 |
| RFTV | -1.364 | 0.744 | -1.833 | 0.067 | NA | NA | NA | NA |
| SAMB | 0.238 | 0.368 | 0.647 | 0.518 | 0.280 | 0.310 | 0.904 | 0.366 |
| TANA | 0.309 | 0.339 | 0.913 | 0.361 | NA | NA | NA | NA |
| TRKX | 0.310 | 0.380 | 0.816 | 0.415 | NA | NA | NA | NA |
| TSVO | 0.403 | 0.365 | 1.104 | 0.270 | 0.557 | 0.310 | 1.796 | 0.073 |

**Table S2.** Community-specific estimates of the slope of the relationship between body mass and dietary grass fraction for large mammalian herbivore communities across central and eastern African communities**,** including only herbivores with dietary grass fraction > 0. Model fit statistics and coefficient estimates account for multiple comparisons.

| Comm-unity | Local diet data | | | | Full community diet data | | | |
| --- | --- | --- | --- | --- | --- | --- | --- | --- |
|  | Slope | Std. Error | *Z*-value | *P*-value | Slope | Std. Error | *Z*-value | *P*-value |
| AMBO | 0.029 | 0.477 | 0.061 | 0.951 | 0.415 | 0.325 | 1.278 | 0.201 |
| ATHI | 0.336 | 0.361 | 0.930 | 0.352 | 0.374 | 0.309 | 1.209 | 0.227 |
| AWSH | 0.778 | 0.537 | 1.449 | 0.147 | 0.746 | 0.470 | 1.587 | 0.112 |
| CHYU | -0.371 | 0.717 | -0.517 | 0.605 | NA | NA | NA | NA |
| ETHR | 0.565 | 0.577 | 0.979 | 0.328 | NA | NA | NA | NA |
| GMBA | -0.508 | 0.646 | -0.786 | 0.432 | 0.483 | 0.338 | 1.426 | 0.154 |
| KBLE | 1.189 | 1.047 | 1.136 | 0.256 | 1.109 | 0.367 | 3.018 | 0.003 |
| KCST | 0.472 | 0.328 | 1.439 | 0.150 | 0.508 | 0.308 | 1.649 | 0.099 |
| KDPO | -0.563 | 0.792 | -0.711 | 0.477 | 0.196 | 0.331 | 0.591 | 0.555 |
| LAIK | 0.308 | 0.339 | 0.909 | 0.364 | NA | NA | NA | NA |
| LEDW | 0.012 | 0.562 | 0.021 | 0.983 | NA | NA | NA | NA |
| LOPE* | NA | NA | NA | NA | 0.775 | 0.686 | 1.130 | 0.258 |
| MAGO | 0.096 | 0.448 | 0.214 | 0.831 | 0.214 | 0.344 | 0.623 | 0.533 |
| MARA | 0.010 | 0.373 | 0.027 | 0.978 | 0.429 | 0.298 | 1.436 | 0.151 |
| MBRO | 0.510 | 0.817 | 0.624 | 0.533 | NA | NA | NA | NA |
| MERU | -1.634 | 0.588 | -2.778 | 0.005 | 0.353 | 0.271 | 1.300 | 0.194 |
| MTKE | 0.406 | 0.414 | 0.981 | 0.327 | 0.451 | 0.343 | 1.314 | 0.189 |
| NAKG | 0.542 | 0.685 | 0.791 | 0.429 | 0.643 | 0.354 | 1.816 | 0.069 |
| RFTV | -1.415 | 0.733 | -1.932 | 0.053 | NA | NA | NA | NA |
| SAMB | 0.245 | 0.360 | 0.681 | 0.496 | 0.289 | 0.305 | 0.946 | 0.344 |
| TANA | 0.322 | 0.332 | 0.969 | 0.332 | NA | NA | NA | NA |
| TRKX | 0.320 | 0.372 | 0.858 | 0.391 | NA | NA | NA | NA |
| TSVO | 0.414 | 0.358 | 1.156 | 0.248 | 0.573 | 0.306 | 1.874 | 0.061 |

*****All LOPE herbivores but one were browsers, such that no slope could be estimated for LOPE when herbivores with dietary grass fraction < 0 were excluded.

**Table S3.** Accounting for spatial autocorrelation in modeling the slope of the relationship between body mass and dietary grass fraction for large mammalian herbivore communities across central and eastern African communities. Different spatial covariance structures were included in global models to assess whether they improved model fit. The model with the lowest AIC_c_ value (depicted in **bold**) was then used as the global model for model selection.

| Table | Herbivore set | Reserve set | Global model | None | Gaussian | Exponential | Spherical |
| --- | --- | --- | --- | --- | --- | --- | --- |
| Table S4 | All herbivores | Full community diet data | Slope ~ SpN + Elephant + PCoA1 + PCoA2 + mean_C_4_ | 0.848 | **-18.254** | -2.953 | -2.383 |
|  |  | Local diet data |  | **48.157** | 50.157 | 50.157 | 50.157 |
|  | Herbivores with dietary grass fraction > 0 | Full community diet data |  | 1.121 | **-11.035** | 0.705 | -1.370 |
|  |  | Local diet data |  | **46.838** | 50.732 | 48.838 | 48.838 |
| Table S5 | All herbivores | Full community diet data | Slope ~ MATemp + MARain + MARain^2^ + Woody_cover + Woody_cover^2^ + TempS +  RainS | 5.574 | **-22.291** | 5.357 | 7.574 |
|  |  | Local diet data |  | **52.962** | 54.962 | 54.962 | 54.962 |
|  | Herbivores with dietary grass fraction > 0 | Full community diet data |  | 14.460 | NA | 14.454 | **11.981** |
|  |  | Local diet data |  | **56.416** | 58.416 | 58.416 | 58.416 |

| Table S6 | All herbivores | Full community diet data | Slope ~ MATemp + MARain + MARain^2^ + Woody_cover + Woody_cover^2^ + TempS +  RainS + SpN + Elephant + PCoA1 + PCoA2 + mean_C_4_ | **-13.944** | NA | -11.944 | -12.088 |
| --- | --- | --- | --- | --- | --- | --- | --- |
|  |  | Local diet data |  | **56.841** | 58.123 | 58.797 | 58.159 |
|  | Herbivores with dietary grass fraction > 0 | Full community diet data |  | **-25.620** | NA | -23.683 | NA |
|  |  | Local diet data |  | **42.328** | 44.108 | 44.313 | 43.923 |

**Table S4.** Predictors of the slope of the relationship between body mass and dietary grass fraction for large mammalian herbivore communities across central and eastern African communities, including community variables only. For each data subset, all plausible models (those with ΔAIC_c_ < 2) are shown (in **bold**), as well as the next best model. Underlined variables arise consistently in plausible models and the direction of the effect is consistent across datasets.

| Herb-ivore set | Res-erve set | Model | R^2^ | *N* | logLik | AIC_c_ | ΔAIC_c_ |
| --- | --- | --- | --- | --- | --- | --- | --- |
| All herbivores | Full comm. | 0.336 – 0.299(Elephant) + 0.014(SpN) | **0.810** | **6** | **15.52** | **-8.541** | **0.000** |
|  |  | 0.335 – 0.314(Elephant) + 0.015(SpN) + 0.055(PCoA2) | 0.822 | 7 | 15.99 | -1.988 | 6.554 |
|  | Local diet data only | 0.096 | **0.070** | **3** | **-18.90** | **45.065** | **0.000** |
|  |  | 0.400 – 0.379(Elephant) | **0.140** | **4** | **-18.00** | **46.220** | **1.155** |
|  |  | 0.527 – 0.544(Elephant) + 0.938(PCoA2) | 0.215 | 5 | -16.95 | 47.421 | 2.356 |
| Herbivores with  dietary grass fraction > 0 | Full comm. | 0.742 – 0.355(Elephant) + 0.908(PCoA2) | **0.727** | **6** | **10.40** | **1.698** | **0.000** |
|  |  | 0.516 + 0.466(PCoA1) | 0.457 | 5 | 5.24 | 6.183 | 4.486 |
|  | Local diet data only | 1.579 – 0.596(Elephant) – 1.994(mean_C_4_) | **0.331** | **5** | **-17.11** | **47.977** | **0.000** |
|  |  | 0.106 | **0.086** | **3** | **-20.55** | **48.433** | **0.457** |
|  |  | 0.863 – 1.509(mean_C_4_) | **0.201** | **4** | **-19.07** | **48.485** | **0.508** |
|  |  | 1.595 – 0.757(Elephant) – 1.768(mean_C_4_) + 1.088(PCoA2) | **0.411** | **6** | **-15.71** | **49.024** | **1.048** |
|  |  | 0.597 – 0.616(Elephant) + 1.333(PCoA2) | **0.271** | **5** | **-18.07** | **49.893** | **1.916** |
|  |  | 0.416 – 0.390(Elephant) | **0.147** | **4** | **-19.79** | **49.939** | **1.962** |
|  |  | 0.108 + 0.819(PCoA2) | 0.141 | 4 | -19.88 | 50.105 | 2.128 |

**Table S5.** Predictors of the slope of the relationship between body mass and dietary grass fraction for large mammalian herbivore communities across central and eastern African communities, including environmental variables only. For each data subset, all plausible models (those with ΔAIC_c_ < 2) are shown (in **bold**), as well as the next best model. Underlined variables arise consistently in plausible models and the direction of the effect is consistent across datasets.

| Herb-ivore set | Res-erve set | Model | R^2^ | *N* | logLik | AIC_c_ | ΔAIC_c_ |
| --- | --- | --- | --- | --- | --- | --- | --- |
| All herbivores | Full community diet data | 0.417 | **0.119** | **4** | **4.00** | **3.995** | **0.000** |
|  |  | 0.501 – 7.38×10^-8^(MARain^2^) | **0.338** | **5** | **6.14** | **4.377** | **0.382** |
|  |  | 0.550 – 1.35×10^-4^(MARain) | **0.299** | **5** | **5.72** | **5.229** | **1.233** |
|  |  | 0.942 – 1.10×10^-7^(MARain^2^) – 5.98×10^-3^ (RainS) | **0.519** | **6** | **8.54** | **5.413** | **1.417** |
|  |  | 0.508 – 3.10×10^-5^(Woody_cover^2^) | **0.279** | **5** | **5.51** | **5.652** | **1.656** |
|  |  | 1.036 – 2.13×10^-4^(MARain) – 6.17×10^-3^ (RainS) | 0.487 | 6 | 8.06 | 6.377 | 2.382 |
|  | Local data only | 0.096 | **0.070** | **3** | **-18.90** | **45.06** | **0.000** |
|  |  | -0.227 + 0.320(TempS) | **0.117** | **4** | **-18.30** | **46.83** | **1.766** |
|  |  | -0.622 + 0.032(MATemp) | 0.104 | 4 | -18.47 | 47.17 | 2.106 |
| Herbivores with  dietary grass > 0 | Full comm. | 0.544 | **0.198** | **4** | **2.31** | **7.374** | **0.000** |
|  |  | 0.848 – 4.39×10^-3^(RainS) | **0.366** | **5** | **4.08** | **8.512** | **1.138** |
|  |  | 1.132 – 0.024(MATemp) | 0.307 | 5 | 3.41 | 9.841 | 2.466 |
|  | Local data only | 0.106 | **0.086** | **3** | **-20.55** | **48.43** | **0.000** |
|  |  | 0.633 – 7.86×10^-3^(RainS) | **0.151** | **4** | **-19.74** | **49.84** | **1.404** |
|  |  | -0.066 + 0.171(TempS) | 0.097 | 4 | -20.42 | 51.19 | 2.752 |

**Table S6.** Predictors of the slope of the relationship between body mass and dietary grass fraction for large mammalian herbivore communities across central and eastern African communities, including both environmental and community variables. For each data subset, all plausible models (those with ΔAIC_c_ < 2) are shown (in **bold**), as well as the next best model. Underlined variables arise consistently in plausible models and the direction of the effect is consistent across datasets.

| Herb-ivore set | Res-erve set | Model | R^2^ | *N* | logLik | AIC_c_ | ΔAIC_c_ |
| --- | --- | --- | --- | --- | --- | --- | --- |
| All herbivores | Full community  diet data | 0.669 – 0.295(Elephant) | **0.269** | **4** | **5.40** | **1.200** | **0.000** |
|  |  | 0.759 + 0.862(PCoA1) – 3.08×10^-7^ (MARain^2^) | **0.458** | **5** | **7.65** | **1.372** | **0.173** |
|  |  | 0.411 | **0.023** | **3** | **3.23** | **1.728** | **0.529** |
|  |  | 0.124 + 0.699(mean_C_4_) | **0.211** | **4** | **4.83** | **2.348** | **1.149** |
|  |  | 0.505 – 8.94×10^-8^(MARain^2^) | **0.175** | **4** | **4.50** | **3.010** | **1.810** |
|  |  | 0.526 – 3.92×10^-5^(Woody_cover^2^) | 0.155 | 4 | 4.31 | 3.377 | 2.177 |
|  | Local diet data only | 0.096 | **0.070** | **3** | **-18.90** | **45.065** | **0.000** |
|  |  | 0.400 – 0.379(Elephant) | **0.140** | **4** | **-18.00** | **46.220** | **1.155** |
|  |  | -0.227 + 0.320(TempS) | **0.117** | **4** | **-18.30** | **46.831** | **1.766** |
|  |  | -0.622 + 0.032(MATemp) | 0.104 | 4 | -18.47 | 47.171 | 2.106 |
| Herbivores with  dietary grass fraction > 0 | Full comm. diet data | 0.518 + 0.600(PCoA1) | **0.414** | **4** | **4.67** | **2.652** | **0.000** |
|  |  | 0.750 + 1.206(PCoA1) – 2.07×10^-7^ (MARain^2^) | **0.553** | **5** | **6.70** | **3.273** | **0.621** |
|  |  | 0.736 + 0.620(PCoA1) – 0.249(Elephant) | **0.543** | **5** | **6.54** | **3.584** | **0.933** |
|  |  | 1.056 – 0.024(SpN) | 0.275 | 4 | 3.07 | 5.861 | 3.209 |
|  | Local diet data only | 3.993 – 3.350(mean_C_4_) – 1.106(Elephant) – 0.022(RainS) – 0.015(Woody_cover) + 0.044(SpN) | **0.691** | **8** | **-8.61** | **44.301** | **0.000** |
|  |  | 3.813 – 3.490(mean_C_4_) – 1.076(Elephant) – 0.021(RainS) – 1.75×10^-4^(Woody_cover^2^) + 0.039(SpN) | **0.682** | **8** | **-8.94** | **44.965** | **0.663** |
|  |  | 3.754 – 3.084(mean_C_4_) – 0.697(Elephant) – 0.016(RainS) – 1.78×10^-4^(Woody_cover^2^) | **0.594** | **7** | **-11.63** | **45.265** | **0.963** |
|  |  | 3.802 – 2.842(mean_C_4_) – 0.668(Elephant) – 0.016(RainS) – 0.014(Woody_cover) | **0.579** | **7** | **-12.03** | **46.057** | **1.756** |
|  |  | 3.721 – 2.845(mean_C_4_) – 0.842(Elephant) – 0.016(RainS) – 1.69×10^-4^(Woody_cover^2^) + 1.016(PCoA2) | **0.663** | **8** | **-9.57** | **46.222** | **1.921** |
|  |  | 2.544 – 2.415(mean_C_4_) – 0.592(Elephant) – 0.011(RainS) | 0.456 | 6 | -14.84 | 47.288 | 2.986 |

**Table S7.** Accounting for spatial autocorrelation in modeling community-averaged dietary grass fraction for large mammalian herbivore communities across central and eastern African communities. Different spatial covariance structures were included in global models to assess whether they improved model fit. The model with the lowest AIC_c_ value (depicted in **bold**) was then used as the global model for model selection.

| Table | Herbivore set | Reserve set | Global model | None | Gaussian | Exponential | Spherical |
| --- | --- | --- | --- | --- | --- | --- | --- |
| Table S8 | All herbivores | Full community diet data | Mean_C_4_ ~  SpN + Elephant + PCoA1 + PCoA2 | 0.079 | -29.894 | -28.901 | -**30.313** |
|  |  | Local diet data |  | 8.219 | -9.489 | -9.452 | **-9.526** |
|  | Herbivores with dietary grass fraction > 0 | Full community diet data |  | -0.090 | -29.404 | -29.029 | **-30.528** |
|  |  | Local diet data |  | 3.184 | -15.788 | -15.759 | **-15.800** |
| Table S9 | All herbivores | Full community diet data | Mean_C_4_ ~ MATemp + MARain + MARain^2^ + Woody_cover+ Woody_cover^2^ + TempS +  RainS | 79.161 | -29.776 | -29.635 | **-29.929** |
|  |  | Local diet data |  | 88.747 | -9.842 | -9.496 | **-10.036** |
|  | Herbivores with dietary grass fraction > 0 | Full community diet data |  | 79.288 | -29.609 | -29.333 | **-29.774** |
|  |  | Local diet data |  | 88.858 | -9.972 | -9.664 | **-10.179** |
| Table S10 | All herbivores | Full community diet data | Mean_C_4_ ~ MATemp + MARain + MARain^2^ + Woody_cover+ Woody_cover^2^ + TempS +  RainS + SpN + Elephant + PCoA1 + PCoA2 | 95.858 | -29.920 | -29.920 | -**29.982** |
|  |  | Local diet data |  | 101.89 | -13.544 | -13.172 | **-13.666** |
|  | Herbivores with dietary grass fraction > 0 | Full community diet data |  | 95.793 | -30.208 | -30.208 | **-30.259** |
|  |  | Local diet data |  | 100.28 | -16.800 | -16.646 | **-16.943** |

**Table S8.** Predictors of community-averaged dietary grass fraction for large mammalian herbivore communities across central and eastern African communities, including community variables only. For each data subset, all plausible models (those with ΔAIC_c_ < 2) are shown (in **bold**), as well as the next best model. Underlined variables arise consistently in plausible models and the direction of the effect is consistent across datasets.

| Herb-ivore set | Ecos-ystem set | Model | R^2^ | *N* | logLik | AIC_c_ | ΔAIC_c_ |
| --- | --- | --- | --- | --- | --- | --- | --- |
| \All herbivores | Full comm. | -3.075 – 0.638(PCoA2) + 5.7×10^-3^(SpN) | **0.733** | **6** | **20.08** | **-17.67** | **0.000** |
|  |  | -2.582 – 0.420(PCoA1) – 0.079(Elephant) | **0.710** | **6** | **19.45** | **-16.41** | **1.260** |
|  |  | 0.186 – 0.516(PCoA2) + 0.016(SpN) – 0.140(Elephant) | **0.821** | **7** | **23.08** | **-16.16** | **1.505** |
|  |  | 0.430 – 0.546(PCoA1) | 0.519 | 5 | 15.66 | -14.66 | 3.004 |
|  | Local diet data only | 0.474 | **-0.04** | **4** | **7.55** | **-4.87** | **0.000** |
|  |  | 0.477 – 0.284(PCoA1) | **0.097** | **5** | **9.20** | **-4.87** | **0.001** |
|  |  | 0.590 – 0.299(PCoA1) – 0.144(Elephant) | **0.227** | **6** | **10.98** | **-4.72** | **0.151** |
|  |  | 0.587 – 0.144(Elephant) | **0.079** | **5** | **8.96** | **-4.40** | **0.469** |
|  |  | 0.476 – 0.325(PCoA2) | **0.079** | **5** | **8.96** | **-4.40** | **0.472** |
|  |  | 0.288 – 0.538(PCoA2) – 0.176(Elephant) + 0.017(SpN) | **0.336** | **7** | **12.74** | **-4.00** | **0.866** |
|  |  | 0.481 – 0.277(PCoA2) – 0.296(PCoA1) | **0.199** | **6** | **10.58** | **-3.91** | **0.958** |
|  |  | 0.269 – 0.572(PCoA2) – 0.011(SpN) | **0.187** | **6** | **10.41** | **-3.56** | **1.308** |
|  |  | 0.494 – 0.202(Elephant) + 7.2×10^-3^(SpN) | 0.140 | 6 | 9.76 | -2.26 | 2.606 |
| Herbivores with  dietary grass fraction > 0 | Full comm. | -3.789 – 0.555(PCoA2) + 4.9×10^-3^(SpN) | **0.776** | **6** | **22.96** | **-23.42** | **0.000** |
|  |  | -4.498 – 0.556(PCoA2) + 3.7×10^-3^ (Elephant) | 0.676 | 6 | 20.21 | -17.92 | 5.502 |
|  | Local diet data only | 0.508 | **-0.03** | **4** | **11.98** | **-13.73** | **0.000** |
|  |  | 0.588 – 0.102(Elephant) | **0.057** | **5** | **13.01** | **-12.49** | **1.239** |
|  |  | 0.509 – 0.189(PCoA2) | 0.024 | 5 | 12.61 | -11.69 | 2.041 |

**Table S9.** Predictors of community-averaged dietary grass fraction for large mammalian herbivore communities across central and eastern African communities, including environmental variables only. For each data subset, all plausible models (those with ΔAIC_c_ < 2) are shown (in **bold**), as well as the next best model. Underlined variables arise consistently in plausible models and the direction of the effect is consistent across datasets.

| Herb-ivore set | Ecos-ystem set | Model | R^2^ | *N* | logLik | AIC_c_ | ΔAIC_c_ |
| --- | --- | --- | --- | --- | --- | --- | --- |
| All herbivores | Full comm. | 0.222 + 0.015(Woody_cover) – 1.97×10^-4^ (Woody_cover^2^) | **0.830** | **6** | **23.45** | **-24.40** | **0.000** |
|  |  | 0.352 + 0.013(Woody_cover) – 1.85×10^-4^ (Woody_cover^2^) – 1.14×10^-3^(RainS) | 0.860 | 7 | 24.94 | -19.88 | 4.527 |
|  | Local diet data only | 0.279 + 0.016(Woody_cover) – 2.16×10^-4^ (Woody_cover^2^) | **0.428** | **6** | **14.44** | **-11.63** | **0.000** |
|  |  | 0.592 – 1.26×10^-7^(MARain^2^) | **0.266** | **5** | **11.58** | **-9.64** | **1.993** |
|  |  | 0.793 – 1.26×10^-7^(MARain^2^) – 2.84×10^-3^ (RainS) | 0.371 | 6 | 13.35 | -9.46 | 2.173 |
| Herbivores with  dietary grass fraction > 0 | Full  comm. | 0.267 + 0.013(Woody_cover) – 1.68×10^-4^ (Woody_cover^2^) | **0.739** | **6** | **21.82** | **-21.13** | **0.000** |
|  |  | 0.155 + 0.018(Woody_cover) – 2.49×10^-4^ (Woody_cover^2^) – 7.79×10^-8^(MARain^2^) | 0.811 | 7 | 24.24 | -18.49 | 2.645 |
|  | Local diet data only | 0.508 | **-0.03** | **4** | **11.98** | **-13.73** | **0.000** |
|  |  | 0.625 – 1.18×10^-3^(RainS) | **0.031** | **5** | **12.69** | **-11.86** | **1.876** |
|  |  | 0.552 – 1.40×10^-5^(Woody_cover^2^) | -0.01 | 5 | 12.27 | -11.00 | 2.728 |

**Table S10.** Predictors of community-averaged dietary grass fraction for large mammalian herbivore communities across central and eastern African communities, including both environmental and community variables. For each data subset, all plausible models (those with ΔAIC_c_ < 2) are shown (in **bold**), as well as the next best model. Underlined variables arise consistently in plausible models and the direction of the effect is consistent across datasets.

| Herb-ivore set | Ecos-ystem set | Model | R^2^ | *N* | logLik | AIC_c_ | ΔAIC_c_ |
| --- | --- | --- | --- | --- | --- | --- | --- |
| All herbivores | Full comm. | 0.222 + 0.015(Woody_cover) – 1.97×10^-4^ (Woody_cover^2^) | **0.830** | **6** | **23.45** | **-24.40** | **0.000** |
|  |  | 0.352 + 0.013(Woody_cover) – 1.85×10^-4^ (Woody_cover^2^) – 1.14×10^-3^(RainS) | 0.860 | 7 | 24.94 | -19.88 | 4.527 |
|  | Local diet data only | 0.282 + 0.016(Woody_cover) – 2.14×10^-4^ (Woody_cover^2^) – 0.333(PCoA2) | **0.542** | **7** | **16.99** | **-12.52** | **0.000** |
|  |  | 0.415 + 0.015(Woody_cover) – 2.05×10^-4^ (Woody_cover^2^) – 0.128(Elephant) | **0.532** | **7** | **16.75** | **-12.03** | **0.490** |
|  |  | 0.279 + 0.016(Woody_cover) – 2.16×10^-4^ (Woody_cover^2^) | **0.428** | **6** | **14.44** | **-11.63** | **0.890** |
|  |  | 0.592 – 1.26×10^-7^(MARain^2^) | 0.266 | 5 | 11.58 | -9.64 | 2.883 |
| Herbivores with  dietary grass fraction > 0 | Full comm. diet data | -3.789 – 0.555(PCoA2) + 4.90×10^-3^(SpN) | **0.776** | **6** | **22.96** | **-23.42** | **0.000** |
|  |  | -4.372 – 0.637(PCoA2) – 0.090(TempS) | **0.767** | **6** | **22.68** | **-22.86** | **0.562** |
|  |  | 0.267 + 0.013(Woody_cover) – 1.68×10^-4^ (Woody_cover^2^) | 0.739 | 6 | 21.82 | -21.13 | 2.290 |
|  | Local diet data only | 0.508 | **-0.03** | **4** | **11.98** | **-13.73** | **0.000** |
|  |  | 0.588 – 0.102(Elephant) | **0.057** | **5** | **13.01** | **-12.49** | **1.239** |
|  |  | 0.625 – 1.76×10^-3^(RainS) | **0.031** | **5** | **12.69** | **-11.86** | **1.876** |
|  |  | 0.509 – 0.189(PCoA2) | 0.024 | 5 | 12.61 | -11.69 | 2.041 |

**Table S11.** Accounting for spatial autocorrelation in modeling intraspecific variation in dietary grass fraction for widespread species within large mammalian herbivore communities across central and eastern African communities. Different spatial covariance structures were included in global models to assess whether they improved model fit. The model with the lowest AIC_c_ value (depicted in **bold**) was then used as the global model for model selection.

| Table | Species | Global model | None | Gaussian | Exponential | Spherical |
| --- | --- | --- | --- | --- | --- | --- |
| Table S12 | *Equus quagga* | Percent_C_4_ ~  SpN + Elephant† + PCoA1 + PCoA2 | 1.607 | -17.439 | -17.439 | **-17.439** |
|  | *Giraffa camelopardalis* |  | 0.534 | **-19.713** | -18.086 | -18.812 |
|  | *Hippopotamus amphibius* |  | 4.455 | **-14.103** | -14.103 | **-**14.103 |
|  | *Kobus ellipsiprymnus* |  | 1.478 | -18.856 | -20.806 | **-20.806** |
|  | *Loxodonta africana*† |  | 6.163 | **-7.517** | -7.353 | -7.300 |
|  | *Phacochoerus africanus* |  | -10.351 | **-37.274** | -36.348 | -35.332 |
|  | *Syncerus caffer* |  | 1.217 | -18.547 | -18.534 | **-18.586** |
| Table S13 | *Equus quagga* | Percent_C_4_ ~ MATemp + MARain + MARain^2^ + Woody_cover + Woody_cover^2^ + TempS +  RainS | 79.059 | NA | -27.264 | **-27.317** |
|  | *Giraffa camelopardalis* |  | 82.363 | **-24.159** | -23.561 | -24.015 |
|  | *Hippopotamus amphibius* |  | 81.262 | **-20.245** | -20.245 | -20.245 |
|  | *Kobus ellipsiprymnus* |  | 79.791 | -19.217 | -19.112 | **-19.222** |
|  | *Loxodonta africana* |  | 89.157 | **-10.098** | -9.359 | -10.005 |
|  | *Phacochoerus africanus* |  | 70.657 | -37.267 | -37.069 | **-37.333** |
|  | *Syncerus caffer* |  | 83.758 | -16.525 | -16.525 | **-16.562** |
| Table S14 | *Equus quagga* | Percent_C_4_ ~ MATemp + MARain + MARain^2^ + Woody_cover + Woody_cover^2^ + TempS +  RainS + SpN + Elephant† + PCoA1 + PCoA2 | 94.392 | NA | -38.150 | **-40.776** |
|  | *Giraffa camelopardalis* |  | 95.493 | -24.359 | -24.264 | **-24.402** |
|  | *Hippopotamus amphibius* |  | 97.196 | **-24.612** | -24.612 | -24.612 |
|  | *Kobus ellipsiprymnus* |  | 93.946 | **-19.486** | -18.227 | -19.227 |
|  | *Loxodonta africana*† |  | 92.994 | -30.246 | -30.239 | **-30.276** |
|  | *Phacochoerus africanus* |  | 92.766 | -34.120 | -34.161 | **-34.251** |
|  | *Syncerus caffer* |  | 100.158 | -16.490 | -16.490 | **-16.490** |

†Note that ‘Elephant’ was not included as a predictor in *Loxodonta africana* models, as elephants were necessarily present at all sites.

**Table S12.** Predictors of intraspecific variation in dietary grass fraction for widespread species within large mammalian herbivore communities across central and eastern African communities, including community variables only. For each data subset, all plausible models (those with ΔAIC_c_ < 2) are shown (in **bold**), as well as the next best model. Underlined variables arise consistently in plausible models and the direction of the effect is consistent across datasets.

| Spec-ies | Model | R^2^ | *N* | logLik | AIC_c_ | ΔAIC_c_ |
| --- | --- | --- | --- | --- | --- | --- |
| *Equus quagga* | 0.808 | **-0.032** | **4** | **12.82** | **-13.64** | **0.00** |
|  | 0.787 – 0.249(PCoA1) | 0.088 | 5 | 13.74 | -10.82 | 2.82 |
| *Giraffa camelop.* | 0.141 | **0.051** | **4** | **16.56** | **-20.68** | **0.00** |
|  | 0.129 – 0.100(PCoA1) | 0.105 | 5 | 16.97 | -16.44 | 4.24 |
| *Hippo. amphibius* | 0.633 | **0.033** | **4** | **14.43** | **-18.01** | **0.00** |
|  | 0.699 – 3.46×10^-3^ (SpN) | 0.082 | 5 | 14.93 | -15.24 | 2.77 |
| *Kobus ellipsi.* | 0.870 | **-0.022** | **4** | **15.44** | **-17.88** | **0.00** |
|  | 0.825 + 0.058(Elephant) | 0.093 | 5 | 16.22 | -13.86 | 4.02 |
| *Loxodonta africana* | 0.257 | **0.098** | **4** | **8.07** | **-3.70** | **0.00** |
|  | 0.258 + 0.217(PCoA1) | 0.172 | 5 | 8.67 | 0.16 | 3.85 |
| *Phaco. africanus* | 0.787 | **0.007** | **4** | **24.56** | **-37.79** | **0.00** |
|  | 0.784 – 0.086(PCoA1) | 0.104 | 5 | 25.43 | -35.41 | 2.37 |
| *Syncerus caffer* | 0.812 – 0.233(PCoA1) | **0.277** | **5** | **15.95** | **-17.29** | **0.00** |
|  | 0.811 | **0.086** | **4** | **13.73** | **-16.59** | **0.69** |
|  | 0.809 – 0.247(PCoA1) – 0.139(PCoA2) | 0.325 | 6 | 16.61 | -14.22 | 3.06 |

**Table S13.** Predictors of intraspecific variation in dietary grass fraction for widespread species within large mammalian herbivore communities across central and eastern African communities, including environmental variables only. For each data subset, all plausible models (those with ΔAIC_c_ < 2) are shown (in **bold**), as well as the next best model. Underlined variables arise consistently in plausible models and the direction of the effect is consistent across datasets.

| Spec-ies | Model | R^2^ | *N* | logLik | AIC_c_ | ΔAIC_c_ |
| --- | --- | --- | --- | --- | --- | --- |
| *Equus quagga* | 0.617 + 8.07×10^-4^(MAR) – 6.73×10^-7^(MAR^2^) | **0.736** | **6** | **23.04** | **-23.58** | **0.000** |
|  | 0.943 – 2.11×10^-7^(MAR^2^) | 0.544 | 5 | 18.94 | -21.21 | 2.369 |
| *Giraffa camelop.* | 0.141 | **0.051** | **4** | **16.56** | **-20.68** | **0.000** |
|  | 0.264 – 1.84×10^-3^(RainS) | **0.284** | **5** | **18.54** | **-19.58** | **1.099** |
|  | 0.050 + 8.50×10^-2^(TempS) | 0.151 | 5 | 17.19 | -17.19 | 3.490 |
| *Hippo. amphibius* | 0.816 – 2.69×10^-3^(RainS) | **0.295** | **5** | **17.43** | **-20.25** | **0.000** |
|  | 1.011 – 3.60×10^-3^(RainS) – 1.70×10^-4^(MAR) | **0.402** | **6** | **18.99** | **-18.99** | **1.262** |
|  | 0.916 – 3.38×10^-3^(RainS) – 7.78×10^-8^(MAR^2^) | 0.370 | 6 | 18.51 | -18.02 | 2.232 |
| *Kobus ellipsi.* | 0.870 | **-0.022** | **4** | **15.44** | **-17.88** | **0.000** |
|  | 0.707 – 3.19×10^-3^(Woody_cover) | 0.171 | 5 | 16.80 | -15.03 | 2.850 |
| *Loxodonta africana* | 0.255 – 2.91×10^-7^(MAR^2^) – 9.93×10^-3^ (Woody_cover^2^) | **0.650** | **6** | **14.69** | **-5.37** | **0.000** |
|  | 0.036 – 5.75×10^-4^(MAR) – 1.10×10^-4^ (Woody_cover^2^) | **0.613** | **6** | **13.99** | **-3.99** | **1.384** |
|  | 0.257 | **0.098** | **4** | **8.07** | **-3.70** | **1.679** |
|  | 0.351– 2.46×10^-7^(MAR^2^) – 6.22×10^-3^ (Woody_cover) | 0.531 | 6 | 12.65 | -1.29 | 4.080 |
| *Phaco. africanus* | 0.835 – 6.13×10^-8^(MAR^2^) | **0.305** | **5** | **27.60** | **-39.74** | **0.000** |
|  | 0.871 – 1.01×10^-4^(MAR) | **0.249** | **5** | **26.94** | **-38.42** | **1.316** |
|  | 0.787 | 0.000 | 4 | 24.50 | -37.67 | 2.064 |
| *Syncerus caffer* | 0.939 – 4.11×10^-5^(Woody_cover^2^) | **0.408** | **5** | **17.85** | **-21.09** | **0.000** |
|  | 0.898 – 9.15×10^-8^(MAR^2^) | **0.404** | **5** | **17.79** | **-20.97** | **0.116** |
|  | 1.002 – 2.08×10^-4^(MAR) | **0.387** | **5** | **17.52** | **-20.43** | **0.657** |
|  | 1.010 – 3.73×10^-3^(Woody_cover) | 0.340 | 5 | 16.82 | -19.02 | 2.066 |

**Table S14.** Predictors of intraspecific variation in dietary grass fraction for widespread species within large mammalian herbivore communities across central and eastern African communities, including both environmental and community variables. For each data subset, all plausible models (those with ΔAIC_c_ < 2) are shown (in **bold**), as well as the next best model. Underlined variables arise consistently in plausible models and the direction of the effect is consistent across datasets.

| Spec-ies | Model | R^2^ | *N* | logLik | AIC_c_ | ΔAIC_c_ |
| --- | --- | --- | --- | --- | --- | --- |
| *Equus quagga* | 0.617 + 8.07×10^-4^(MAR) – 6.73×10^-7^(MAR^2^) | **0.736** | **6** | **23.04** | **-23.58** | **0.000** |
|  | 0.943 – 2.11×10^-7^(MAR^2^) | 0.544 | 5 | 18.94 | -21.21 | 2.369 |
| *Giraffa camelop.* | 0.138 | **0.014** | **4** | **16.30** | **-20.15** | **0.000** |
|  | 0.261 – 1.80×10^-3^(RainS) | **0.256** | **5** | **18.27** | **-19.04** | **1.106** |
|  | 0.043 + 9.08×10^-2^(TempS) | 0.101 | 5 | 16.94 | -16.38 | 3.769 |
| *Hippo. amphibius* | 0.816 – 2.69×10^-3^(RainS) | **0.295** | **5** | **17.43** | **-20.25** | **0.000** |
|  | 1.012 – 3.60×10^-3^(RainS) – 1.70×10^-4^(MAR) | **0.402** | **6** | **18.99** | **-18.99** | **1.262** |
|  | 0.916 – 3.38×10^-3^(RainS) – 7.78×10^-8^(MAR^2^) | 0.370 | 6 | 18.51 | -18.02 | 2.232 |
| *Kobus ellipsi.* | 0.870 | **-0.022** | **4** | **15.44** | **-17.88** | **0.000** |
|  | 0.708 – 3.19×10^-3^(Woody_cover) | 0.171 | 5 | 16.80 | -15.03 | 2.850 |
| *Loxodonta africana* | 0.250 – 2.92×10^-7^(MAR^2^) – 9.82×10^-3^ (Woody_cover^2^) | **0.646** | **6** | **14.62** | **-5.24** | **0.000** |
|  | 0.033 – 5.75×10^-4^(MAR) – 1.10×10^-4^ (Woody_cover^2^) | **0.608** | **6** | **13.90** | **-3.79** | **1.447** |
|  | 0.257 | **0.095** | **4** | **8.04** | **-3.64** | **1.594** |
|  | 0.351– 2.47×10^-7^(MAR^2^) – 1.16×10^-4^ (Woody_cover^2^) + 0.370(PCoA1) | 0.780 | 7 | 17.93 | -3.19 | 2.044 |
| *Phaco. africanus* | 0.835 – 6.13×10^-8^(MAR^2^) | **0.305** | **5** | **27.60** | **-39.74** | **0.000** |
|  | 0.871 – 1.01×10^-4^(MAR) | **0.249** | **5** | **26.94** | **-38.42** | **1.316** |
|  | 0.787 | 0.000 | 4 | 24.50 | -37.67 | 2.064 |
| *Syncerus caffer* | 0.939 – 4.12×10^-5^(Woody_cover^2^) | **0.406** | **5** | **17.83** | **-21.04** | **0.000** |
|  | 0.898 – 9.17×10^-8^(MAR^2^) | **0.403** | **5** | **17.78** | **-20.94** | **0.099** |
|  | 1.003 – 2.08×10^-4^(MAR) | **0.386** | **5** | **17.51** | **-20.40** | **0.641** |
|  | 1.010 – 3.74×10^-3^(Woody_cover) | 0.338 | 5 | 16.79 | -18.97 | 2.072 |


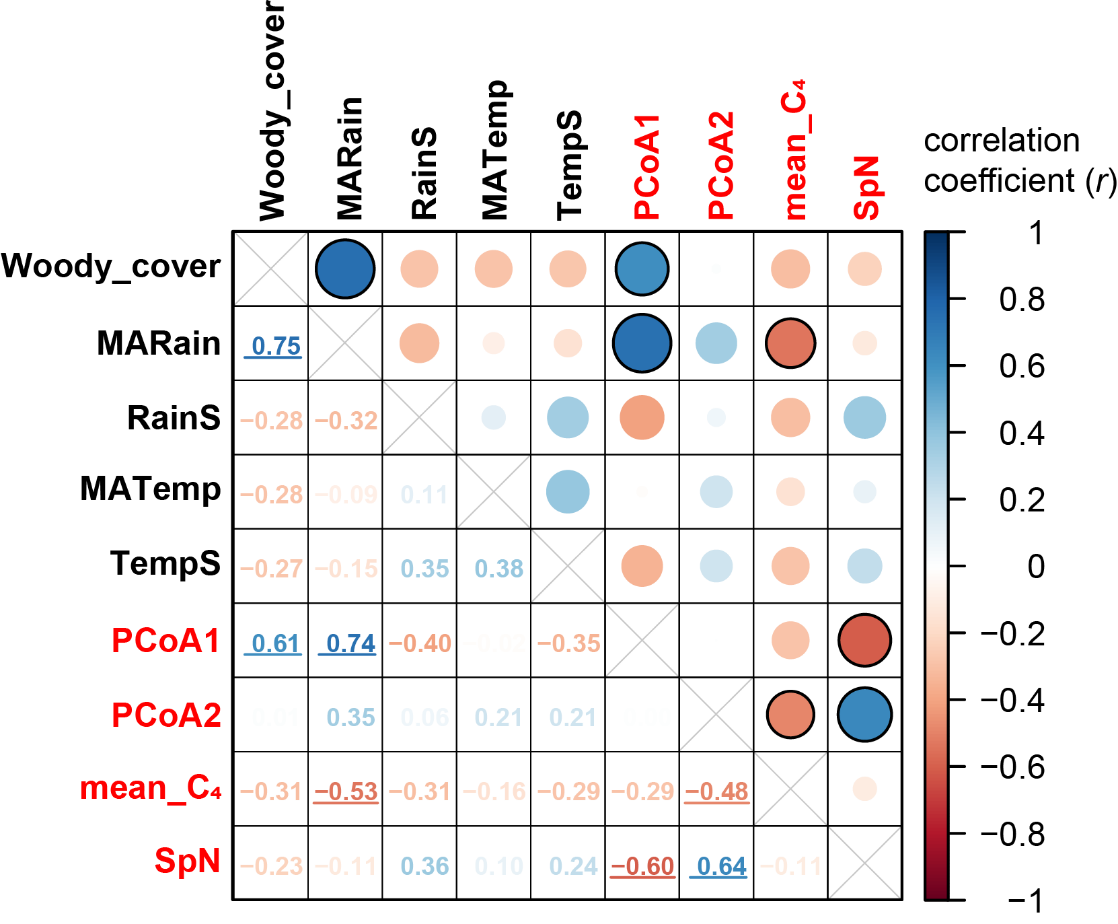


**Figure S1.** Correlation between predictor variables included in model selection. Variable names are colored according to whether they are environmental (*black*) or community (*red*) variables. Above the diagonal, circle size and color correspond to the magnitude, direction, and significance of correlations between all variables, where *larger and darker* colored circles reflect correlation coefficients of greater magnitude, *blue* colored circles represent positive correlation coefficients, *red* colored circles represent negative correlation coefficients, and circles with *black outlines* represent significant correlations (*P* < 0.05). Below the diagonal, numbers reflect the correlation coefficients of all variable combinations, where number color and underlining represents the direction and significance of correlations respectively: *darker* colored numbers reflect correlation coefficients of greater magnitude, *blue* colored numbers represent positive correlation coefficients, *red* colored numbers represent negative correlation coefficients, and *underlined* numbers represent significant correlations (*P* < 0.05). Only one of any variable pair that covaried significantly with one another (significantly correlated variables with correlation coefficient *r* > 0.5) were included in any given model.
